# Glycosylphosphatidylinositol Mannosyltransferase Ⅰ Protects Chinese Giant Salamander, *Andrias davidianus,* against Iridovirus

**DOI:** 10.3390/ijms23169009

**Published:** 2022-08-12

**Authors:** Jingjing Zhang, Yanlin Dai, Yuding Fan, Nan Jiang, Yong Zhou, Lingbing Zeng, Yiqun Li

**Affiliations:** 1 Yangtze River Fisheries Research Institute, Chinese Academy of Fishery Sciences, Wuhan 430223, China; 2 National Demonstration Center for Experimental Fisheries Science Education, Shanghai Ocean University, Shanghai 201306, China

**Keywords:** Chinese giant salamander, AdGPI-MT-I, iridovirus, antiviral activities

## Abstract

Glycosylphosphatidylinositol mannosyltransferase I (GPI-MT-I) is an essential glycosyltransferase of glycosylphosphatidylinositol-anchor proteins (GPI-APs) that transfers the first of the four mannoses in GPI-AP precursors, which have multiple functions, including immune response and signal transduction. In this study, the GPI-MT-I gene that regulates GPI-AP biosynthesis in *Andrias davidianus* (*AdGPI-MT-I*) was characterized for the first time. The open reading frame (ORF) of *AdGPI-MT-I* is 1293 bp and encodes a protein of 430 amino acids that contains a conserved PMT2 superfamily domain. *AdGPI-MT-I* mRNA was widely expressed in the tissues of the Chinese giant salamander. The mRNA expression level of *AdGPI-MT-I* in the spleen, kidney, and muscle cell line (GSM cells) was significantly upregulated post Chinese giant salamander iridovirus (GSIV) infection. The mRNA expression of the virus major capsid protein (MCP) in AdGPI-MT-I-overexpressed cells was significantly reduced. Moreover, a lower level of virus MCP synthesis and gene copying in AdGPI-MT-I-overexpressed cells was confirmed by western blot and ddPCR. These results collectively suggest that GSIV replication in GSM cells was significantly reduced by the overexpression of the AdGPI-MT-I protein, which may contribute to a better understanding of the antiviral mechanism against iridovirus infection.

## 1. Introduction

Glycosylphosphatidylinositol-anchor proteins (GPI-APs) are conserved post-translational modification proteins that bind different proteins to the cell surface and have multiple functions, including signal transduction, immune response, and biosynthesis [1]. In mammalian cells, GPI-APs have different functions as hydrolase enzymes, receptors, adhesion molecules, complement regulatory proteins, and other immune-related proteins [2,3]. In fungi, the GPI biosynthetic pathway is necessary for cell wall biosynthesis and the survival of yeast cells [4]. In the protozoa *Plasmodium falciparum* and *Trypanosoma brucei*, GPI-APs are the predominant membrane attachment of cell surface proteins and play a role in the pro-inflammatory immune response to parasitic infection [5,6]. Therefore, GPI synthesis could be exploited as a target for antifungal or antiparasitic agents. Moreover, GPI-APs have been used as protein transfer vectors to regulate host immune responses in mammalian cells. Lipid raft-resident GPI-APs have been reported to regulate leukocyte adhesion, polarization, and motility in both integrin-dependent and -independent manners [7]. Many T cell surface proteins are attached to the cell membrane by a GPI anchor, which may be involved in cell signaling, protein targeting, or protein release. The purified GPI-AP MHC class I molecule HLA-A2.1 delivers a hepatitis B virus antigen peptide to the surface of a cytotoxic T cell target, which activates specific T cells [8]. The biosynthesis of GPI-APs occurs in the ER by the sequential addition of sugars to the phosphatidylinositol through the coordinated activity of several proteins, among which glycosylphosphatidylinositol mannosyltransferase I (GPI-MT-I) is one of the most essential mannosyltransferases [9,10].

GPI-APs play a crucial role during pathogen invasion, and the essential mannosyltransferase GPI-MT-I during its biosynthetic pathway might be a reasonable target for the development of antipathogen drugs [9,11]. As an essential enzyme for adding mannose on the glycosylphosphatidyl group, protozoan mannosyltransferase (GPI-14) is a key enzyme in the biosynthesis of lipophosphoglycan (LPG) and glycoinositolphospholipids (GIPLs), which play important roles in the parasite infectious cycle [12]. Ribeiro et al. proposed that the overexpression of GPI-14 in both proflagellate and anflagellate Leishmania brasiliensis protects the parasite against trivalent antimony, indicating that GPI-14 has significant potential as a target for new leishmaniasis treatment alternatives [13]. Recent studies showed that virus-like particles containing GPI-anchored mucose-associated epithelial chemokine (CCL28) vaccine preparations elicited high and sustained levels of long-term virus-specific antibodies and reduced viral load and inflammatory responses during immunization with murine influenza H3N2 viruses and mucosal antibody-induced responses [14]. In humans, the GPI-AP biosynthesis pathway is highly conserved [15]. Nevertheless, the function of GPI-MT-I in lower vertebrates such as amphibians remains unclear and needs further investigation.

The Chinese giant salamander, *Andrias davidianus*, is the largest extant amphibian species in the world and is classified as a critically endangered species by the International Union for Conservation of Nature in China [16]. Because of its unique evolutionary status, it is a good specimen for studying biological evolution and species diversity [17,18]. In recent years, the outbreak of the Chinese giant salamander iridovirus (GSIV) brought great losses to the breeding industry, and there is no medical treatment for this disease. GSIV, a member of the genus Ranavirus and belonging to the iridovirus family, is a double-stranded DNA virus with icosahedral symmetry and a diameter of approximately 140 nm [19,20,21]. The GSIV infection of Chinese giant salamanders causes anorexia, lethargy, skin ulceration, and tissue necrosis, and the mortality rate of giant salamanders infected with iridovirus is more than 90% [22]. Electron microscopy revealed that hexagonal or round virus particles mostly existed in the cytoplasmic region in spleen and kidney tissue samples. Both spleen and kidney tissues of GSIV-infected salamanders showed varying degrees of damage, with many splenocytes, enlarged necrotic nuclei, proliferative lymphoid nodules, and vacuolar degeneration detected in the spleen. The vacuolar degeneration and necrosis of renal hematopoietic tissue cells and glomeruli were detected in the kidney tissues [19,23]. In EPC cells infected with GSIV, the virus mostly aggregated in the cytoplasmic and nucleus breaking regions and crossed the cell membrane in a budding manner to form an envelope [19]. In order to explore more effective drugs and understand the mechanism of virus inhibition, it is necessary to further study the characteristics of GPI-MT-I.

In this study, the characteristics and function of *GPI-MT-I* (*AdGPI-MT-I*) in the Chinese giant salamander were investigated. The results indicated that *AdGPI-MT-I* played an inhibitory role during GSIV infection, representing the first report on the involvement of *GPI-MT-I* in antiviral immunity in amphibians. This study may increase our understanding of the regulatory mechanisms during iridovirus infection.

## 2. Results

### 2.1. Sequence Characterization of AdGPI-MT-I

**Figure 1:**
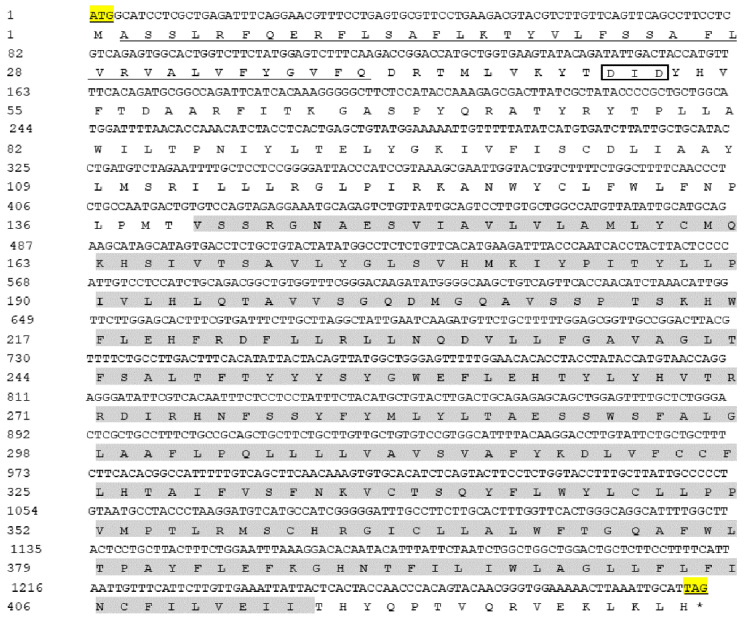
Nucleotide and deduced amino acid sequence analysis of open reading frame (ORF) of *AdGPI-MT-I*. Yellow highlights indicate start codons and stop codons, and asterisk indicates the termination of amino acids. The grey shading indicates the PMT2 super family domain, and the underlined part is the predicted signal peptides. The DXD motif is marked with a box (□).

**Figure 2:**
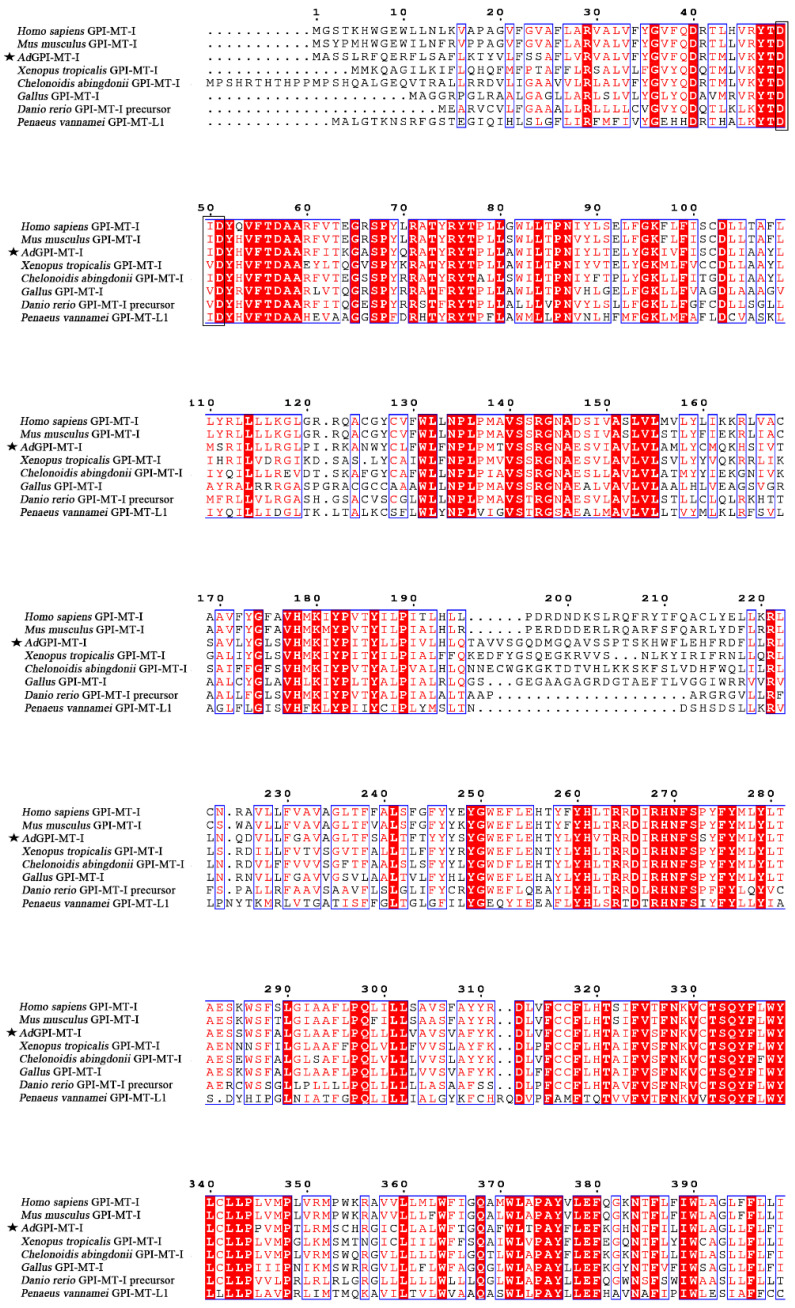
Multiple sequence alignment by Clustal W of *GPI-MT-I* gene encoding proteins from different species. Consensus residues are shaded in red; residues that are ≥75% identical among the aligned sequences are identified with red letters. The AdGPI-MT-I is marked with a pentagram (★), and the DXD motif is marked with a black box (□). The sequences used are listed in Table 1.

**Figure 3:**
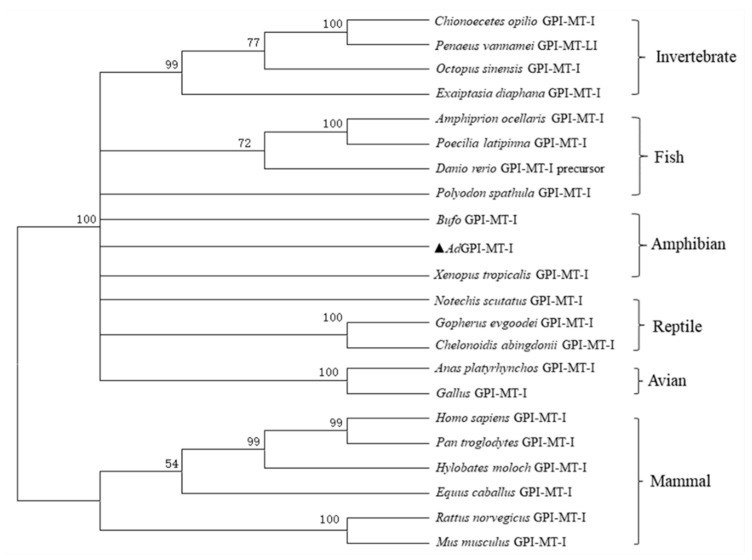
Phylogenetic relationships between GPI-MT-I genes in different species. Amino acid sequences of GPI-MT-I genes were deduced from GenBank, and the phylogenetic tree was constructed with the neighbor-joining method in MEGA7.0 software. Numbers beside the internal branches indicate bootstrap values based on 1000 replications. AdGPI-MT-I is highlighted with a triangle symbol (▲).

The open reading frame sequence of the *AdGPI-MT-I* gene is 1293 bp and encodes a protein of 430 amino acid residues. The molecular weight of the protein encoded by *AdGPI-MT-I* is about 50 kDa, and the theoretical isoelectric point is 9.016. Similar to other GPI-MT-I homologues, the AdGPI-MT-I protein possesses a conserved dolichyl-phosphate-mannose-protein mannosyltransferase (PMT2) superfamily domain (140–414 aa) containing a mannosyltransferase domain (PIG-M) and a GPI transamidase subunit PIG-U domain (Figure 1). Multiple alignment indicated that AdGPI-MT-I shared 63–98% overall sequence identity with the GPI-MT-I homologues of *Homo sapiens*, *Mus musculus*, *Xenopus tropicalis*, *Danio rerio*, and *Gallus* and *Penaeus vannamei* (Figure 2). The GPI-MT-I proteins from various organisms share a DXD motif, which is circled in a black box in Figure 2. In addition, a phylogenetic tree of the amino acid sequences of AdGPI-MT-I and GPI-MT-I in different species was constructed using the neighbor-joining method to reveal their phylogenetic relationships. As shown in Figure 3, AdGPI-MT-I was closest to *Xenopus tropicalis* GPI-MT-I and *Bufo* GPI-MT-I among the examined species (Figure 3).

**Table 1:** GenBank accession numbers of GPI-MT-I for phylogenetic analysis and multiple sequence alignments.

Taxonomy	Species/Gene	Accession Number
Mammal		
	*Homo sapiens* GPI-MT-I	NP_660150.1
	*Pan troglodytes* GPI-MT-I	NP_001233402.1
	*Hylobates moloch* GPI-MT-I	XP_032009911.1
	*Rattus norvegicus* GPI-MT-I	NP_077058.1
	*Mus musculus* GPI-MT-I	NP_080510.1
	*Equus caballus* GPI-MT-I	XP_005610027.1
Avian		
	*Gallus gallus* GPI-MT-I	NP_001026693.2
	*Anas platyrhynchos* GPI-MT-I	XP_005023671.2
Reptile		
	*Gopherus evgoodei* GPI-MT-I	XP_030410133.1
	*Chelonoidis abingdonii* GPI-MT-I	XP_032648289.1
	*Notechis scutatus* GPI-MT-I	XP_026547789.1
Amphibian		
	*Xenopus tropicalis* GPI-MT-I	NP_001008120.1
	*Bufo bufo* GPI-MT-I	XP_040289781.1
Fish		
	*Polyodon spathula* GPI-MT-I	XP_041101136.1
	*Danio rerio* GPI-MT-I	NP_956684.1
	*Amphiprion ocellaris* GPI-MT-I	XP_023121236.1
	*Poecilia latipinna* GPI-MT-I	XP_014911736.1
Invertebrate		
	*Octopus sinensis* GPI-MT-I	XP_029633767.1
	*Exaiptasia diaphana* GPI-MT-I	KXJ16443.1
	*Penaeus vannamei* GPI-MT-I like	XP_027225082.1
	*Chionoecetes opilio* GPI-MT-I	KAG0721514.1

### 2.2. Expression Patterns of AdGPI-MT-I in Tissues and Cells

**Figure 4:**
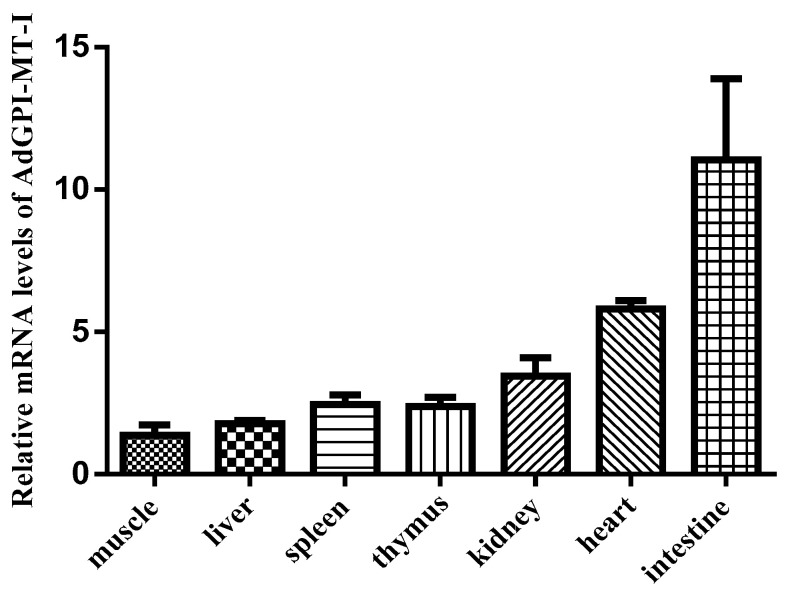
The mRNA transcription levels of *AdGPI-MT-I* in six healthy Chinese giant salamander tissues including muscle, liver, spleen, thymus, kidney, heart, and intestine. The mRNA expression of *AdGPI-MT-I* was detected by quantitative real-time PCR (qRT-PCR), which was normalized to β-actin. The expression level in muscle was set as 1. Data are the means of three independent assays and are presented as means ± SD.

**Figure 5:**
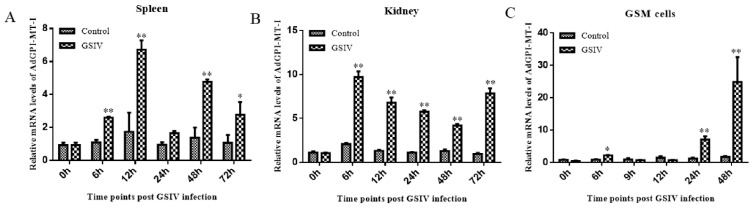
Expressions of *AdGPI-MT-I* in the spleen (**A**), kidney (**B**), and GSM cells (**C**) at indicated times post GSIV infection. The mRNA expression level of *AdGPI-MT-I* was determined by quantitative real-time PCR (qRT-PCR), with six salamanders included in each time period. For the convenience of comparison, the expression level in the control at 0 h normalized to β-actin was set as 1. Error bars indicate the mean ± SD (*n* = 3). ** *p* < 0.01, * *p* < 0.05. Six salamanders were included in each time period.

The qRT-PCR test showed that *AdGPI-MT-I* was widely expressed in the examined tissues, with the highest level in the intestine and heart; an intermediate level in the thymus, kidney, and spleen; and the lowest level in the liver and muscle (Figure 4). To further understand the changes in the expression of *AdGPI-MT-I* during virus infection, the expression level of *AdGPI-MT-I* in the spleen, kidney, and GSM cells was detected at 0, 6, 12, 24, 48, and 72 h post GSIV infection. Compared with the control group, the mRNA expression level of *AdGPI-MT-I* in the spleen was slightly increased at 6 h (2.5-fold, *p* < 0.01), peaked at 12 h (seven-fold, *p* < 0.01), decreased to the initial level at 24 h, and increased significantly at 48 h (five-fold, *p* < 0.01) and 72 h (2.6-fold, *p* < 0.05) after GSIV infection (Figure 5A). Meanwhile, the mRNA expression level of *AdGPI-MT-I* in the kidney was obviously increased at 6 h (*p* < 0.01); after that, it gradually decreased until the upward trend appeared again at 72 h after GSIV infection (Figure 5B). In the GSM cells, the *AdGPI-MT-I* mRNA expression level in GSIV-infected cells was induced at 6 h (2.2-fold, *p* < 0.05) and significantly upregulated at 24 h (7-fold, *p* < 0.01) compared with the control, then reached its peak at 48 h (24-fold, *p* < 0.01) after infection (Figure 5C).

### 2.3. Subcellular Localization of AdGPI-MT-I

**Figure 6:**
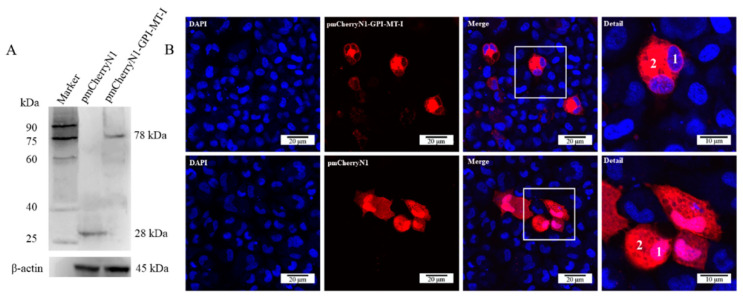
(**A**) Western blot was used to verify the overexpression of the AdGPI-MT-I protein in GSM cells. GSM cells were transfected with pmCherryN1 vector or pmCherryN1-AdGPI-MT-I plasmid DNA for 48 h and then harvested for western blot. β-actin was used as an internal reference. (**B**) Confocal microscopy was used to observe the subcellular localization of the AdGPI-MT-I protein in GSM cells (scale bar, 20 μm). Nuclei were stained with DAPI. The nucleus and cytoplasm are marked with the numbers 1 and 2, respectively.

The molecular weight of eukaryotic plasmid pmCherryN1-AdGPI-MT-I was predicted to be 78 kDa, while that of pmCherryN1 was 28 kDa, and the western blot result confirmed the successful expression of pmCherryN1-AdGPI-MT-I in the GSM cells (Figure 6A). Confocal microscopy showed that the main expression site of the AdGPI-MT-I protein was in the cytoplasm (Figure 6B).

### 2.4. Antiviral Effect of AdGPI-MT-I in GSM Cells

**Figure 7:**
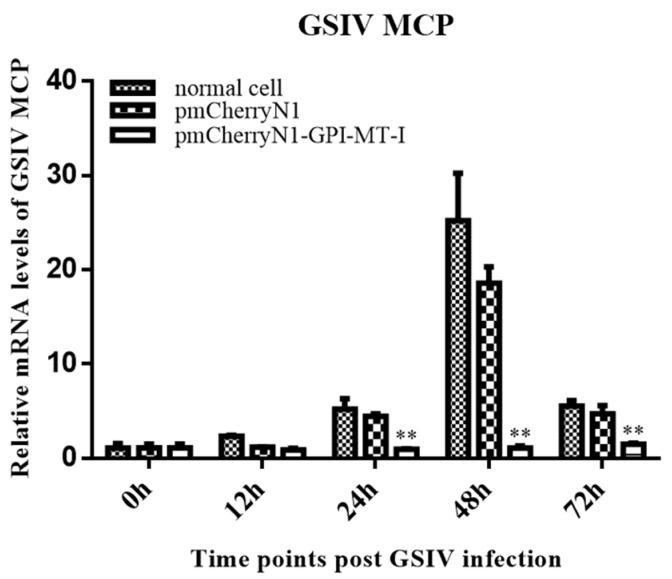
Effect of overexpression of *AdGPI-MT-I* on mRNA expression of major capsid protein (MCP) of GSIV detected by quantitative real-time PCR (qRT-PCR). The normal GSM cells and cells transfected with the empty vector plasmid were set as the control group, and the experimental group was transfected with pmCherryN1-AdGPI-MT-I plasmid for 48 h. At 0, 12, 24, 48, and 72 h after GSIV infection (MOI = 0.1), cell samples were collected for RNA extraction, and the transcription levels of the MCP gene were detected by qRT-PCR. All the experiments were repeated three times. Data are the means of three independent assays and presented as means ± SD; ** *p* < 0.01.

In the pmCherryN1-AdGPI-MT-I-plasmid-transfected GSM cells, the mRNA expression level of GSIV MCP significantly decreased at 24 h (4.5-fold, *p* < 0.01), 48 h (20-fold, *p* < 0.01), and 72 h (5-fold, *p* < 0.01) compared with the normal cells and cells transfected with the empty vector plasmid (Figure 7).

**Figure 8:**
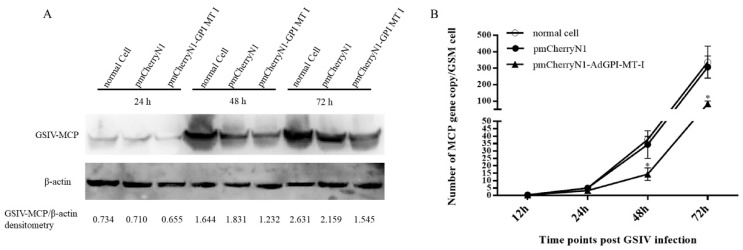
(**A**) Western blot analysis of MCP protein expression and grayscale quantitative analysis. Western blot was used to detect protein synthesis after GSIV infection at different times, and mouse anti-MCP monoclonal antibody and anti-β-actin monoclonal antibodies were used as primary antibodies, respectively. HRP-tagged antimouse IgG was used as a secondary antibody. β-actin was used as internal control. The gray values of GSIV-MCP and β-actin protein bands were quantitatively analyzed by ImageJ software. (**B**) Digital droplet PCR (ddPCR) was used to detect the MCP gene copy number of GSIV in GSM cells. After transfection of pmCherryN1-AdGPI-MT-I plasmid with GSM cells for 48 h, the copy number of the MCP gene at 0, 12, 24, 48, and 72 h after GSIV infection was detected by ddPCR. Error bars indicate the mean ± SD (*n* = 3). The asterisks indicate significant difference (* *p* < 0.05) between treated and control groups.

To further quantify the inhibitory effect of AdGPI-MT-I on virus proliferation, western blot was used to detect MCP protein expression, and digital droplet PCR (ddPCR) was used to detect the virus copies during GSIV infection. As shown in Figure 8A, after the overexpression of AdGPI-MT-I in GSM cells for 48 h, the GSM cells were infected with GSIV at 24 h, 48 h, and 72 h, and the MCP protein level was detected by western blot. Gray scale quantitative analysis showed that compared with the cells transfected with pmCherryN1 plasmid and normal cells, the level of GSIV MCP protein in the experimental group was significantly reduced (Figure 8A). Moreover, compared with the control groups, the ddPCR results showed that the MCP gene copy number of GSIV was significantly reduced after infection with GSIV for 48 h and 72 h (Figure 8B).

## 3. Discussion

GPI-APs are a series of integral membrane proteins present on the cell surface that have various functions, including as hydrolytic enzymes, receptors, adhesion molecules, protease inhibitors, complement regulators, and prions. Previous studies showed that the steps in the GPI-AP assembly pathway and the various enzymes that perform these steps are highly conserved, but with slight differences. The GPI moiety of GPI-APs contains the conserved core glycan with the structure of EtNP6-Manα2-Manα6-(EtNP)2-Manα4-GINα-myoIno-Plipid (Man, Mannose). GPI-MT-I, a mannosyltransferase required for the addition of the first mannose, is often used as a target for specific inhibitors of GPI-AP biosynthesis [24,25]. In mammals, GPI-MT-I contains a GPI transamidase subunit PIG-U domain and a mannosyltransferase domain (PIG-M), which together form a PMT2 superfamily domain [9,26,27]. PIG-U is thought to represent the fifth subunit in this complex and may be involved in the recognition of either the GPI attachment signal or the lipid portion of GPI [28,29]. The DXD motif in the PIG-M domain is thought to be involved in the binding of manganese ions, which are required for enzymes to bind to nucleotide sugar substrates, and the DXD motif is functionally essential, suggesting that the first mannose transfer occurs in the luminal side of the ER [30]. In this study, we determined that Chinese giant salamander GPI-MT-I shares the conserved PMT2 superfamily domain and contains a DXD motif. Sequence alignment and phylogenetic tree analysis showed that the *AdGPI-MT-I* gene had high homology with the *GPI-MT-I* gene of other species. The above analysis indicated that *AdGPI-MT-I* is a member of the glycosylphosphatidylinositol mannitol transferase I (GPI-MT-I) family.

GPI-Aps exist in a wide range of organisms, including mammals, protozoans, fungi, insects, and plants. Hundreds of human proteins, such as cell surface receptors, enzymes, cell adhesion molecules, and mutated protective antigens, are anchored by glycosylphosphatidylinositol (GPI) structures, as they lack transmembrane domains [31]. The mammalian GPI-APs consist of three mannoses attached to acylated glucosamine-(acyl) phosphatidylinositol (GlcN-(acyl) PI) to form mannose (Man) (3)-GlcN-(acyl) PI, in which GPI-MT-I transfers the first mannose to GlcN-(acyl) PI [32]. In our study, *AdGPI-MT-I* was detected in all tissues, which implied universal GPI-AP synthesis in the Chinese giant salamander. The products of the GPI-AP biosynthetic pathway appear to be widely distributed in the intracellular intimal system [33]. GPI anchoring is a post-translational modification which arises on the endoplasmic reticulum (ER) membrane, undergoes several remodeling steps in the ER and the Golgi apparatus, and is finally transported to the plasma membrane (PM). Previous studies showed that the biosynthesis of GlcN-(acyl) PI occurred on the cytoplasmic side, and the luminal location of the functionally important DXD motile was one of the pieces of evidence supporting the luminal location of the GPI mannose transfer reaction [25,30]. Subcellular localization revealed that AdGPI-MT-I was mainly distributed in the cytoplasm of GSM cells, which further suggested its mannose-adding function. In diseases involving GPI-APs, pathology can induce changes in GPI-AP expression or properties. At present, it is difficult to elucidate the nature of the causative agent, and only the correlation between abnormal GPI-APs and the pathological state can be observed [34]. Notably, as a key factor in intracellular pathogen recognition, toll-like receptor 7 was expressed in the kidney and spleen at 6 h and 48 h after GSIV infection, respectively [35]. The transcript levels of the innate immune-related myxovirus-resistance and 2′-5′-oligoadenylate synthetase genes were also significantly upregulated in the spleen and kidney at 6 h after infection, followed by a significant upregulation in the type I IFN expression at 12 h [36,37,38]. In this study, we noted that *AdGPI-MT-I* was significantly upregulated in the spleen and kidney tissues of Chinese giant salamanders at 6 h after GSIV infection, indicating that *AdGPI-MT-I* might contribute to the eradication of the pathogen by regulating the anchoring of the GPI-APs involved in GSIV invasion. The relative expression of different immune genes thought to be affected by GPI-MT-I will be investigated in further studies.

In clinical studies, gene defects involved in the synthesis and processing of GPI-APs can result in a variety of genetic diseases, particularly affecting immune response and signal transduction [39]. GPI-APs in various parasitic protozoans can mediate cellular immune responses through toll-like family members of pattern recognition receptors [40]. Furthermore, as a crucial enzyme in GPI-AP biosynthesis, GPI-AP glycosyltransferases may be one of the potential targets during pathogen infection [41]. In the present study, to demonstrate whether AdGPI-MT-I has antiviral properties, GSM cells were transfected with pmCherryN1-AdGPI-MT-I plasmid and then infected with GSIV. *AdGPI-MT-I* overexpression significantly downregulated GSIV MCP gene transcription in GSM cells, and consistent results were detected for GSIV MCP gene copies and protein expression, suggesting that GPI-MT-I might play a key role in the immune response of giant salamanders against GSIV infection. We speculate that the overexpression of AdGPI-MT-I altered the anchoring process of the GPI-APs, which was beneficial for virus elimination and suppressed virus propagation. Due to the lack of sufficient quantities of pure anchors and anchored proteins, it is difficult to study the characteristics and relationships among various glycosyltransferases during the synthesis of GPI-APs. Although the genes involved in the biosynthesis of GPI-APs are well characterized, the regulation of GPI biosynthesis during virus infection remains unclear and needs further investigation.

In conclusion, this study identified an *AdGPI-MT-I* gene from Chinese giant salamanders and explored its cellular expression and antiviral function. The results showed that the gene was mainly located in the cytoplasm. The overexpression of AdGPI-MT-I has an inhibitory effect on GSIV replication by restraining the viral transcription, protein expression, and viral load. This study is the first to demonstrate the antiviral function of GPI-associated enzymes in amphibians, providing a new insight for further study of the antiviral mechanisms of the Chinese giant salamander.

## 4. Materials and Methods

### 4.1. Animals, Cells, and Virus

Chinese giant salamanders were obtained from the Yangtze River Fishery Research Institute of the Chinese Academy of Fishery Sciences in Wuhan, China. Before testing, all the salamanders were kept in tanks for one month at 20 °C and fed with fresh fish meal once a day. PCR detection confirmed that these animals were free of GSIV. All animal procedures were conducted in accordance with the recommendations in the guidelines of the Care and Use of Laboratory Animals Monitoring Committee of Hubei Province, China. The Chinese giant salamander muscle cell line (GSM cells) was generously provided by Professor Qi-ya Zhang (Institute of Water Biology, Chinese Academy of Sciences). GSM cells were cultured in a T_25_ cell culture flask (Corning, Corning, NY, USA) with a density of 6 × 10^6^ cells/mL, with 5 mL M199 medium (Hyclone, Logan, UT, USA) containing 10% fetal bovine serum (FBS) (Hyclone, Logan, UT, USA). The incubator temperature was 25 °C. The GSIV was originally isolated and identified from diseased Chinese giant salamanders by our laboratory [19].

### 4.2. DNA Extraction, RNA Extraction, and cDNA Preparation

Viral DNA was extracted using a viral DNA kit (Omega, Norcross, GA, USA) according to the manufacturer’s protocol, and the TCID_50_/mL of the GSIV was measured using the Reed and Muench methods [42]. Total RNA was extracted using TRIzol reagent (Thermo Scientific, Waltham, MA, USA), and residual DNA traces were removed using DNase I (TaKaRa, Taejin, Japan). After extraction, the integrity and purity of the RNA were detected by agarose gel electrophoresis and a NanoDrop One spectrophotometer (Thermo Fisher Scientific). Finally, a TRANS reverse transcription kit (TransGen, Beijing, China) was used for reverse transcription. The cDNA and RNA templates of all samples were stored at −80 °C for later use.

### 4.3. cDNA Cloning of AdGPI-MT-I and Bioinformatic Analysis of Sequences

The cDNA sequence of *AdGPI-MT-I* was acquired from the transcriptome database of Chinese giant salamanders in our lab. A pair of primers, AdGPI-MT-I-ORF-F1 and AdGPI-MT-I-ORF-R1, was designed to specifically amplify the AdGPI-MT-I ORF sequence using Primer Premier 5 software (Table 2). Initial denaturation was performed at 94 °C for 4 min, followed by 35 cycles of 94 °C for 30 s, annealing at 59 °C for 30 s, and extension at 72 °C for 1 min. After the thermal cycle, PCR amplification was performed by extension at 72 °C for another 10 min to complete PCR amplification. The following operations were performed according to the manufacturer’s instructions. PCR specific products were collected, followed by purification of the products with a Gel Extraction kit (Omega Bio-tek, Norcross, GA, USA), and then the products were ligated and inserted into the pMD19-T vector (TaKaRa, Taejin, Japan) and sequenced at TsingKe Biotechnology Company for confirmation (TsingKe, Beijing, China).

The BLAST program of the National Center of Biotechnology Information (available online: http://www.ncbi.nlm.nih.gov/blast (accessed on 11 March 2021)) was used to search sequences. The signal peptide was analyzed online using SignalP-4.0 Server (available online: http://www.cbs.dtu.dk/services/SignalP-4.0/ (accessed on 13 October 2021)). Multiple amino acid sequence alignments were generated with Clustal W (available online: http://www.ebi.ac.uk/Tools/clustalw/ (accessed on 15 October 2021). Conserved domains were predicted by SMART (available online: http://smart.emblheidelberg.de/ (accessed on 15 October 2021)), and the conserved residues were shaded using DNAMAN (V6). The phylogenetic tree was constructed by MEGA 7.0 [43] using the neighbor-joining (NJ) algorithm. Genbank accession numbers of the selected sequences were obtained from the National Center of Biotechnology Information (NCBI) and are shown in Table 1.

### 4.4. Detection of AdGPI-MT-I Distribution in Tissues by Quantitative Real-Time PCR (qRT-PCR)

Six healthy giant salamanders were euthanized with tricaine methanesulfonate MS222 (100 mg/L, sigma). Various tissues, including muscle, liver, spleen, kidney, thymus, heart, and intestinal, were collected. cDNA templates were collected according to the above method for qRT-PCR analysis.

### 4.5. The Effects of GSIV on the AdGPI-MT-I Expression in Tissues and GSM Cells

Seventy-two healthy salamanders with negative GSIV-detection results were randomly divided into two groups of thirty-six animals, with six salamanders for each time period. The animals were observed daily for signs of infection and mortality after an intraperitoneal injection of 200 μL of GSIV (1.0 × 10^7.8^ TCID_50_/mL) per animal and an equal amount of PBS (Hyclone, Logan, UT, USA) in the control group. Furthermore, spleens and kidneys were collected at different time points (0, 6, 12, 24, 48, and 72 h) post injection. In addition, GSM cells were cultured overnight in 6-well plates (2 × 10^6^ cells/mL), and the experimental group was stimulated with 1 mL of GSIV (1.0 × 10^7.8^ TCID_50_/mL) diluted 100-fold for 2 h, followed by the addition of 500 μL of M199 medium containing 2% fetal bovine serum (Hyclone, Logan, UT, USA). The control group was given the same treatment with serum-free M199 medium (Hyclone, Logan, UT, USA). Cell samples were collected at different time points (0, 6, 9, 12, 24, and 48 h) after GSIV infection (MOI of 0.1), transcribed into cDNA as described above, and characterized in vitro for the expression of the *AdGPI-MT-I* gene after GSIV infection. Three wells were tested for each time period.

In order to test the *AdGPI-MT-I* expression in vivo and in vitro after infection with GSIV, qRT-PCR was performed with primers AdGPI-MT-I-rqF and AdGPI-MT-I-rqR (Table 2). The β-actin of the Chinese giant salamander (GenBank accession number: HQ822274) was amplified as the internal control gene for cDNA normalization (Table 2). The qRT-PCR primers were verified to be specific, with only one target PCR product. The melt curve of the qRT-PCR primers had a single peak, and the threshold period (CT) was less than 30. The qRT-PCR test was performed on a Rotor-Gene 6000 Real-Time PCR system (Qiagen, Dusseldorf, Germany) using a 2 × TB Green Fast qPCR Mix (TaKaRa, Taejin, Japan). The qRT-PCR program consisted of 1 cycle at 95 °C for 5 min, followed by 40 cycles at 95 °C for 20 s, 60 °C for 20 s, and 72 °C for 20 s. The qRT-PCR mixture consisted of 10 μL Power 2 × TB Green Fast qPCR mixture (TaKaRa, Taejin, Japan), 0.8 μL of each primer (10 M), 2 μL of diluted cDNA samples, and 6.4 μL of sterile H_2_O. Relative qRT-PCR gene expression analysis was performed using the 2^−4ΔΔCT^method, and all the experiments were repeated three times [44].

### 4.6. AdGPI-MT-I Expression Plasmid Construction, Subcellular Localization, and Western Blot Confirmation

A pair of specific primers, AdGPI-MT-I-ORF-F2 and AdGPI-MT-I-ORF-R2, with *Bam*H I and *Xho*I (shown in Table 2) restriction sites added were designed to amplify the cDNA of *AdGPI-MT-I*. The specific PCR product was purified as mentioned above, inserted into the pMD19-T vector (TaKaRa, Taejin, Japan), and then ligated into the pmCherryN1 expression vector (pmCherryN1-AdGPI-MT-I) by double digestion using *Bam*H I and *Xho*I. After successful ligation, the product was removed and expanded with an Endo free Plasmid Mini Kit (Omega, Norcross, GA, USA) to extract the recombinant plasmid pmCherryN1-AdGPI-MT-I and then sequenced (TsingKe, Beijing, China).

GSM cells were seeded into 6-well plates at a density of 2 × 10^6^ cells/mL according to the above method and cultured for 24 h until a cell monolayer of approximately 70–90% confluence was formed. Then, 500 μL of M199 (Hyclone, Logan, UT, USA) medium containing 5 μg of pmCherryN1-AdGPI-MT-I or pmCherryN1 plasmid and 10 μL of lipofectamine™ 3000 (Invitrogen, Carlsbad, CA, USA) was introduced into each well of cells according to the manufacturer’s instructions. Twelve hours after transfection, fresh M199 (Hyclone, Logan, UT, USA) medium containing 10% FBS (Hyclone, Logan, UT, USA) was replaced. After 48 h of transfection, the cell medium was removed, and the GSM cells were washed three times with PBS (Hyclone, Logan, UT, USA), fixed with 4% paraformaldehyde for 20 min, and washed three times again with PBS (Hyclone, Logan, UT, USA) before transfection with 6-diamidino-2-phenyl-ndole (DAPI) (Solarbio, Beijing, China) to stain the cell nuclei. Finally, culture dishes (Biosharp, Anhui, China) were washed with PBS (Hyclone, Logan, UT, USA) and examined by confocal microscopy (Olympus, Tokyo, Japan).

After 48 h of transfection with the above method, cells were harvested and the expression of pmCherryN1-AdGPI-MT-I was detected via western blot with anti-mCherry-Tag mouse polyclonal antibodies (#AE002, ABclonal, Wuhan, China). Meanwhile, the expression of β-actin was detected by western blot with anti-β-actin mouse polyclonal antibodies (#12262, CST, Danvers, MA, USA).

### 4.7. Antiviral Activity of AdGPI-MT-I in GSM Cells

The GSM cells were cultured under the same conditions as described above and transfected with pmCherryN1-AdGPI-MT-I and pmCherryN1 plasmid, respectively, as before. After 48 h of transfection, three parallel samples were collected at 0, 12, 24, 48, and 72 h post infection (MOI of 0.1). The control group was given the same amount of PBS (Hyclone, Logan, UT, USA). cDNA templates were collected according to the above method for qRT-PCR analysis. qRT-PCR was performed to quantify the major capsid protein (MCP) of the virus using the primer MCP-rqF/MCP-rqR, as shown in Table 2. The amplification was performed by a Rotor-Gene 6000 Real-Time PCR system (Qiagen, Dusseldorf, Germany). Relative qRT-PCR gene expression analysis was performed using the 2^−ΔΔCT^ method, with the β-actin gene used as the internal control gene for cDNA normalization [44]. All the experiments were repeated three times.

Moreover, after 48 h of transfection with the above method, three parallel samples were collected at 0, 24, 48, and 72 h after GSIV infection (MOI of 0.1). Protein samples were processed as follows: First, proteins were resolved with 12% SDS-PAGE gel at around 120 v for 90 min. Then, samples were transferred onto 0.45 μm pore nitrocellulose membrane using a semi-dry blotter (Bio-Rad, Hercules, CA, USA); washed with TBST for 5 min; and blocked with TBST containing 5% skimmed milk for 2 h at 37 °C. Next, the membrane was incubated with our laboratory’s anti-MCP mouse monoclonal antibodies (1:1000) and anti-β-actin mouse monoclonal antibodies (#12262, CST, Danvers, MA, USA) at 4 °C overnight. The membranes were washed three times with TBST; incubated with alkaline horseradish peroxidase-conjugated anti-mouse IgG (#58802, CST, Danvers, MA, USA) for 2 h at room temperature; and finally washed three times with TBST. Then, the washed PVDF membranes were incubated with Clarity^TM^ Western ECL substrate (Bio-Rad, Hercules, CA, USA) for 5 min and exposed through a gel imaging system (Bio-Rad, Hercules, CA, USA). EasySee Western Marker (25–90 kDa) (TransGen, Beijing, China) was used in the experiments.

### 4.8. Digital Droplet PCR (ddPCR) Detection of Major Capsid Protein (MCP) Gene Copies of GSIV

The GSM cells were cultured under the same conditions as above and transfected with pmCherryN1-AdGPI-MT-I and pmCherryN1 plasmid, respectively, as before. After 48 h of transfection, three parallel samples were collected at 0, 24, 48, and 72 h (MOI of 0.1) after GSIV infection. Viral DNA was isolated using a Viral DNA Kit (OMEGA, Norcross, GA, USA) and stored at −80 °C. Amplification was performed using a QX200™ Droplet digital PCR™ system (Bio-Rad, Hercules, CA, USA). The ddPCR-related primers are listed in Table 2, and the amplification procedure consisted of 1 cycle at 95 °C for 5 min; 40 cycles of 30 s at 95 °C and 1 min at 60.8 °C; and 1 cycle at 98 °C for 10 min. The amplification system was as follows: 2 μL diluted DNA samples; 10 μL ddPCR Supermix for Probes (Bio-Rad, Hercules, CA, USA); 2 μL of each primer (10 M); 0.5 μL of specific probe (10 M); and 4 μL sterile H_2_O.

### 4.9. Statistical Analysis

Statistical summarization of qRT-PCR and ddPCR data was performed using Graphpad Prism 6.01 software (Version X, La Jolla, CA, USA), and statistical significance between samples was analyzed by one-way analysis of variance (ANOVA). The western blot results were quantified in grayscale using ImageJ software [45]. All data are presented in mean ± standard deviation format. Error bars indicate mean ± SD (*n* = 3). Values of *p* < 0.05 were considered statistically significant differences, and values of *p* < 0.01 were considered extreme differences.

## Figures and Tables

**Table 2:** The relevant primers used in this study.

Application	Primer Name	Nucleotide Sequence (5′-3′)
PCR		
	AdGPI-MT-I-ORF-F1	ATGGCATCCTCGCTGAGATTTCA
	AdGPI-MT-I-ORF-R1	CTAATGCAATTTAAGTTTTTCCACC
	AdGPI-MT-I-ORF-F2	CCGGATCCATGGCATCCTCGCTGAGATTTCA
	AdGPI-MT-I-ORF-R2	GCCTCGAGCTAATGCAATTTAAGTTTTTCCACC
	GSIV-F	GACTTGGCCACTTATGAC
	GSIV-R	GTCTCTGGAGAAGAAGAA
qRT-PCR		
	AdGPI-MT-I-rqF	TTACTCCCCATTGTCCTCCA
	AdGPI-MT-I-rqR	GTGTTCCAAAAACTCCCAGC
	MCP-rqF	GCGGTTCTCACACGCAGTC
	MCP-rqR	ACGGGAGTGACGCAGGTGT
	β-actin-rqF	TGAACCCAAAAGCCAACCGAGAAAAGAT
	β-actin-rqR	TACGACCAGAGGCATACAGGGACAGGAC
ddPCR		
	GSIV MCP-F	GCGGTTCTCACACGCAGTC
	GSIV MCP-R	ACGGGAGTGACGCAGGTGT
	GSIV probe	FAM + AGCCGACGGAAGGGTGTGTGAC + TAMARA

## Data Availability

Not applicable.

## Abbreviations

GPI-MT-I	Glycosylphosphatidylinositol mannosyltransferase I
GPI-APs	Glycosylphosphatidylinositol-anchor proteins
GSIV	Chinese giant salamander iridovirus
GSM cells	Chinese giant salamander muscle cell line
MCP	Chinese giant salamander iridovirus major capsid protein
